# Early estimates of nirsevimab immunoprophylaxis effectiveness against hospital admission for respiratory syncytial virus lower respiratory tract infections in infants, Spain, October 2023 to January 2024

**DOI:** 10.2807/1560-7917.ES.2024.29.6.2400046

**Published:** 2024-02-08

**Authors:** Mónica López-Lacort, Cintia Muñoz-Quiles, Ainara Mira-Iglesias, F Xavier López-Labrador, Beatriz Mengual-Chuliá, Carlos Fernández-García, Mario Carballido-Fernández, Ana Pineda-Caplliure, Juan Mollar-Maseres, Maruan Shalabi Benavent, Francisco Sanz-Herrero, Matilde Zornoza-Moreno, Jaime Jesús Pérez-Martín, Santiago Alfayate-Miguelez, Rocío Pérez Crespo, Encarnación Bastida Sánchez, Ana Isabel Menasalvas-Ruiz, Mª Cinta Téllez-González, Samuel Esquiva Soto, Carlos Del Toro Saravia, Iván Sanz-Muñoz, José María Eiros, Vanesa Matías Del Pozo, Marina Toquero-Asensi, Eliseo Pastor-Villalba, José Antonio Lluch-Rodrigo, Javier Díez-Domingo, Alejandro Orrico-Sánchez

**Affiliations:** 1 Vaccine Research Department, Fisabio-Public Health, Valencia, Spain; 2 CIBERESP, Instituto de Salud Carlos III, Madrid, Spain; 3 Virology Laboratory, Fisabio-Public Health, Valencia, Spain; 4 Department of Microbiology and Ecology, Medical School, University of Valencia, Valencia, Spain; 5 Hospital General Universitario de Castellón, Castellón, Spain; 6 Medicine Department, Universidad CEU Cardenal Herrera, Castellón de la Plana, Spain; 7 Hospital Universitario Dr. Peset, Valencia, Spain; 8 Hospital Universitario La Fe, Valencia, Spain; 9 Hospital Marina Baixa, Alicante, Spain; 10 Hospital General Universitario, Valencia, Spain; 11 Murcia Health Council, Murcia, Spain; 12 Hospital Clínico Universitario Virgen de la Arrixaca, Murcia, Spain; 13 Hospital General Universitario Santa Lucía, Murcia, Spain; 14 Hospital Rafael Méndez, Murcia, Spain; 15 National Influenza Centre, Valladolid, Spain; 16 Hospital Clínico, Valladolid, Spain; 17 Dirección General de Salud Pública, Valencia, Spain; 18 Catholic University of Valencia, Valencia, Spain; * These authors contributed equally to this article and share first authorship.

**Keywords:** Respiratory Syncytial Virus, RSV, immunoprophylaxis, effectiveness, long-acting monoclonal antibodies, nirsevimab

## Abstract

The monoclonal antibody nirsevimab was at least 70% effective in preventing hospitalisations in infants with lower respiratory tract infections (LRTI) positive for respiratory syncytial virus (RSV) in Spain (Oct 2023–Jan 2024), where a universal immunisation programme began late September (coverage range: 79–99%). High protection was confirmed by two methodological designs (screening and test-negative) in a multicentre active surveillance in nine hospitals in three regions. No protection against RSV-negative LRTI-hospitalisations was shown. These interim results could guide public-health decision-making.

Respiratory syncytial virus (RSV) is the leading cause of acute lower respiratory tract infections (LRTI) in children. Nirsevimab (Beyfortus, AstraZeneca/Sanofi Pasteur), a novel monoclonal antibody against RSV, has shown promising potential in preventing paediatric RSV infections [1,2], with clinical trials showing 77.3% efficacy in preventing RSV-LRTI hospital admission in healthy infants born at term or preterm [3]. Nirsevimab immunisation also showed an 83% reduction in RSV-related hospitalisations in infants in a real-world clinical trial setting [4] and an impact on the reduction of severity of RSV-related hospitalisations [5]. However, estimates of direct effectiveness have not yet been published. In late September 2023, Spain introduced the universal RSV prophylaxis into its national immunisation programme [6] for all infants born from 1 April 2023. Here, we provide the early estimates of the effectiveness of nirsevimab against hospital admission for RSV-LRTI in infants (< 9 months old) in three autonomous regions of Spain.

## Active hospital surveillance of respiratory syncytial virus

We conducted a multicentre hospital-based active surveillance in nine hospitals located in three autonomous regions (five hospitals in Valencia, three in Murcia and one in Valladolid, a province of Castilla y León) in Spain. All participating hospitals routinely tested hospitalised patients for respiratory infections. The population during the data collection period consisted of all infants eligible for immunisation with nirsevimab during their first RSV season (born from 1 April 2023, n = 15,676 infants, representing 6.4% of the entire Spanish infant population eligible for immunisation). The surveillance period lasted from 1 October 2023 to between 31 December 2023 and 10 January 2024, depending on the hospital (Table 1). All infants admitted with LRTI were included. 

**Table 1:** Nirsevimab immunisation coverage and infant hospitalisations with lower respiratory tract infections by hospital, three regions in Spain, October 2023–January 2024 (n = 166 hospitalisations)

Hospitals by region	End of data collection period^a^	Infants < 9 months of age (n)	Population-based immunisation coverage (%)^b^	LRTI (n)	RSV-LRTI (n)	RSV-LRTI immunised (%)	Negative RSV-LRTI (n)	Negative RSV-LRTI immunised (%)	LRTI per 1,000 infants	RSV-LRTI per 1,000 infants
**Valencia **		7,191	89.8	52	36	72.2	16	81.3	7.2	5.0
Hospital General Universitario de Castellón	31 Dec 2023	1,387	87.5	12	9	33.3	3	100.0	8.7	6.5
Hospital Universitari Politecnic La Fe	10 Jan 2024	2,144	93.6	13	9	33.3	4	75.0	6.1	4.2
Consorcio Hospital General Universitario Valencia	6 Jan 2024	1,621	91.9	12	11	9.1	1	100.0	7.4	6.8
Hospital Universitario Dr Peset	10 Jan 2024	1,19	91.0	9	4	100.0	5	80.0	7.6	3.4
Hospital de la Marina Baixa de la Vila Joiosa	31 Dec 2023	849	78.7	6	3	66.7	3	66.7	7.1	3.5
**Murcia **		7,449	88.9	107	52	50.0	55	83.6	14.4	7.0
Hospital Rafael Méndez	31 Dec 2023	1,263	89.6	8	3	66.7	5	80.0	6.3	2.4
Hospital General Universitario Santa Lucía	31 Dec 2023	1,555	88.6	39	20	60.0	19	94.7	25.1	12.9
Hospital Clínico Universitario Virgen de la Arrixaca^c^	8 Jan 2024	4,631	88.8	60	29	41.4	31	77.4	13..0	6.3
**Valladolid**										
Hospital Clínico Valladolid	31 Dec 2023	1,036	98.6	7	7	57.1	0	0	6.8	6.8

LRTI: lower respiratory tract infections; RSV: respiratory syncytial virus.

^a^ Start of data collection date was 1 October 2023.

^b^ Immunisation coverage data in Valencia are up to 12 Jan 2024, in Murcia up to 5 Jan 2024, in Valladolid up to 15 Jan 2024.

^c^ At Hospital Clínico Universitario Virgen de la Arrixaca, 11 RSV-LRTI cases were confirmed by antigen testing. The remaining cases in all hospitals were confirmed by PCR.

Physician-confirmed LRTI admissions were ascertained through a retrospective review of hospital records (Murcia and Valladolid) or the database of the Valencia Hospital Network for the Study of Influenza and other respiratory viruses (VAHNSI). The VAHNSI network annually conducts a prospective active hospital-based surveillance where all infants admitted to one of the five hospitals with severe acute respiratory infection (SARI) are tested for RSV by multiplex RT-PCR [7], given parental consent. The European Centre for Disease Prevention and Control (ECDC) clinical SARI case definition [8] used in the VAHNSI project included any hospitalisation with respiratory symptoms with an onset within 14 days prior to admission.

A description of hospitalisations of infants with LRTI is shown in Table 2. Overall, 166 admissions for LRTI were included. Of the admitted infants, 95 were positive for RSV and 73 were aged 0–3 months. Murcia and Valencia reported the first RSV cases in week 40 and 41, respectively, and 75% (66/88) of cases from these regions were concentrated in weeks 47–52 2023. Valladolid had their first RSV cases in week 47 and most cases (5/7) were concentrated between weeks 48–52. All three regions still had RSV cases in week 1 2024, indicating that the season had not yet concluded. Among all 95 RSV cases, 56 (59%) had been immunised (Table 2). 

**Table 2:** Characteristics of infant hospitalisations with lower respiratory tract infections and nirsevimab immunisation status, stratified by respiratory syncytial virus (RSV) PCR results, three regions in Spain, October 2023–January 2024 (n = 166 admissions)

Characteristics	Murcia (n = 107)		Valencia (n = 52)		Valladolid (n = 7)
	Negative RSV-LRTI	RSV-LRTI	Negative RSV-LRTI	RSV-LRTI	RSV-LRTI
	n = 55	n = 52	n = 16	n = 36	n = 7
Age at hospitalisation (months)					
0	15	9	1	3	1
1	14	13	1	11	2
2	9	10	6	8	2
3	4	8	3	5	1
4	4	5	1	3	1
5	5	1	2	0	0
6	2	4	0	3	0
7	2	1	1	2	0
8	0	1	1	1	0
0–3	42	40	11	27	6
Nirsevimab immunisation					
Yes	46	26	13	26	4
Age at immunisation (months)					
0	29	16	4	16	3
1	5	2	3	4	0
2	9	3	2	1	1
3	1	2	1	0	0
4	2	2	1	2	0
5	4	1	1	1	0
6	0	0	1	2	0
7	0	0	0	0	0
8	1	0	0	0	0
Unknown	4	26	3	10	3
Month of hospitalisation					
Oct 2023	14	0	2	0	0
Nov 2023	14	11	7	7	2
Dec 2023	19	36	5	23	3
Jan 2024	8	5	2	6	2

LRTI: lower respiratory tract infections; RSV: respiratory syncytial virus.

Nirsevimab population-based coverage ranged between 78.7% and 98.6%, depending on the hospital (Table 1). Infants were considered immunised from the date of administration of nirsevimab (ranging from 25 September 2023 to 14 December 2023). Immunisation status and population-based coverage data were obtained from regional vaccination registries (VIS, VACUSAN, and REVA in Valencia, Murcia, and Valladolid, respectively). The number of LRTI hospitalisations according to aetiology (RSV-positive and -negative), age (in months) and immunisation status is appended in Supplementary Figure S1.

## Estimating effectiveness of nirsevimab immunoprophylaxis 

### Screening method

Effectiveness of nirsevimab immunoprophylaxis by region was assessed by the screening method, in which the proportion of infants immunised with nirsevimab among RSV-LRTI hospitalised cases was compared to the proportion of immunised infants in the corresponding region/hospital catchment area. Effectiveness was estimated as (1−odds ratio (OR)) x 100, with a 95% credible interval (CI) using Bayesian binomial regression, adjusted by hospital. Nirsevimab effectiveness was 69.3% (95% CI: 36.4–86.2), 86.9% (95% CI: 77.1–92.9) and 97.0% (95% CI: 87.7–99.6) in Valencia, Murcia and Valladolid, respectively (Table 3).

**Table 3:** Effectiveness of nirsevimab against hospitalisation in infants by the screening method and test-negative design, three regions in Spain, October 2023–January 2024 (n = 166 admissions)

Method	RSV-LRTI (n = 95)		Negative RSV-LRTI (n = 71)		
	(1−OR) x 100	95% CI	(1−OR) x 100	95% CI	
**Screening**					
Murcia	86.9	77.1 to 92.9	27.5		−47.3 to 66.2
Valencia	69.3	36.4 to 86.2	19.6		−180.8 to 82.3
Valladolid	97.0	87.7 to 99.6	NA		
Pooled data	84.4	76.8 to 90.0	32.4		−27.5 to 63.4^a^
**Test-negative design**					
Pooled data	70.2	38.3 to 88.5^a^	NA		

CI: credible interval; LRTI: lower respiratory tract infection; NA: not available; OR: odds ratio; RSV: respiratory syncytial virus.

^a^ Only data from Murcia and Valencia were considered because there were no negative RSV-LRTI in Valladolid.

The similarities in the methodology used to collect LRTI hospitalisations and to routinely test RSV aetiology among the three regions allowed for pooling estimates. To accomplish this, a Bayesian binomial regression, considering the hospital as a random effect to address unspecified features of each setting, was developed. Pooled nirsevimab effectiveness against RSV-LRTI admissions was 84.4% (95% CI: 76.8–90.0).

### Test-negative design

Using a test-negative design (TND), data from Valencia and Murcia were pooled to estimate nirsevimab effectiveness against RSV-LRTI admissions. The immunisation odds of infants testing RSV-positive by PCR (n = 77) were compared with infants testing negative (n = 71) to estimate nirsevimab effectiveness by a Bayesian logistic regression considering hospital as a random effect. Overall nirsevimab effectiveness was 70.2% (95% CI: 38.3–88.5) (Table 3).

### Sensitivity analysis

In a sensitivity analysis, we used the screening method to estimate the effectiveness of nirsevimab to prevent LRTI admissions negative for RSV, as 19.6% (95% CI: −180.8 to 82.3) and 27.5% (95% CI: −47.3 to 66.2) in Valencia and Murcia, respectively. Pooled effectiveness including both regions was 32.4% (95% CI: −27.5 to 63.4). Data from Valladolid could not be used in the sensitivity analysis because of the lack of RSV-negative cases (Table 3).

## Discussion

This study provides an early estimation of the nirsevimab effectiveness in preventing RSV-LRTI hospitalisation in infants 3 months after its introduction into the Spanish national immunisation programme in a real-world setting. The strength of the study lies in the availability to pool data from hospital surveillance in three Spanish regions using a similar methodology for detecting RSV-hospitalised cases, and the simultaneous application of two independent designs (screening and TND), including a negative outcome. Our estimates indicate that nirsevimab effectiveness was greater than 70% in infants under 9 months of age who were candidates to receive the immunisation. The lack of protection in the RSV-negative hospitalisations supports the estimates.

Apart from the efficacy shown in randomised clinical trials, nirsevimab treatment was recently demonstrated to elicit an 83% reduction in RSV-related hospitalisations in infants under conditions that approximated real-world settings [4]. In line with these results, our pooled estimates showed that nirsevimab was between 70% and 84% effective in preventing hospitalisations for RSV-LRTI in infants. Spain was among the first countries in Europe to introduce nirsevimab into a publicly funded national immunisation programme [6]. During the first 3 months, nirsevimab coverage in all eligible cohorts in the participating regions was around 90% on average. The active surveillance of respiratory infections performed in all nine participating hospitals allowed us to provide these estimates with two widely used methods to estimate vaccine effectiveness [9-12].

These results predict a possible important impact of the nirsevimab immunisation programme on the high RSV disease burden that, according to a 2022 study from five countries in Europe, accounts for one RSV-related hospitalisation per 56 infants in high-income settings [13]. In Spain, 3.2 per 100 infants under 6 months of age are hospitalised for an RSV bronchiolitis [14]. The immunisation with nirsevimab is expected to alleviate the high pressure on the healthcare systems, especially during winter periods.

The limitations of this study include considerable variation in reported effectiveness estimates because of differences in RSV circulation, hospital admission policies, sparse LRTI hospitalisations, prophylaxis coverage and case definition among hospitals and regions. However, the potential variability among hospitals was controlled for in the models. In addition, a more sensitive case definition was used in Valencia, where only severe acute respiratory infection admissions with onset of symptoms < 14 days [8] and parental consent were included, which could have led to some case loss in comparison to the other regions. Nevertheless, this issue probably did not interfere in the estimation of effectiveness. Given the short time since the implementation of the nirsevimab immunisation programme (3 months), the small number of cases prevented us from evaluating regional estimates of efficacy using TND and further evaluations later in season are warranted.

## Conclusions

These early real-world data provide evidence that nirsevimab protects infants against hospitalisation for RSV-associated LRTI. While further confirmation with more studies is required, these interim results could provide valuable insights for public health decision-making. 

## Supplementary Data

144000 Supplement

## References

[1] Griffin MP Yuan Y Takas T Domachowske JB Madhi SA Manzoni P Single-dose nirsevimab for prevention of RSV in preterm infants. N Engl J Med. 2020;383(5):415-25. 10.1056/NEJMoa1913556 32726528

[2] Hammitt LL Dagan R Yuan Y Baca Cots M Bosheva M Madhi SA Nirsevimab for prevention of RSV in healthy late-preterm and term infants. N Engl J Med. 2022;386(9):837-46. 10.1056/NEJMoa2110275 35235726

[3] Simões EAF Madhi SA Muller WJ Atanasova V Bosheva M Cabañas F Efficacy of nirsevimab against respiratory syncytial virus lower respiratory tract infections in preterm and term infants, and pharmacokinetic extrapolation to infants with congenital heart disease and chronic lung disease: a pooled analysis of randomised controlled trials. Lancet Child Adolesc Health. 2023;7(3):180-9. 10.1016/S2352-4642(22)00321-2 36634694 PMC9940918

[4] Drysdale SB Cathie K Flamein F Knuf M Collins AM Hill HC Nirsevimab for prevention of hospitalizations due to RSV in infants. N Engl J Med. 2023;389(26):2425-35. 10.1056/NEJMoa2309189 38157500

[5] Ernst C Bejko D Gaasch L Hannelas E Kahn I Pierron C Impact of nirsevimab prophylaxis on paediatric respiratory syncytial virus (RSV)-related hospitalisations during the initial 2023/24 season in Luxembourg. Euro Surveill. 2024;29(4):2400033. 10.2807/1560-7917.ES.2024.29.4.2400033 38275017 PMC10986653

[6] Martinón-Torres F Mirás-Carballal S Durán-Parrondo C . Early lessons from the implementation of universal respiratory syncytial virus prophylaxis in infants with long-acting monoclonal antibodies, Galicia, Spain, September and October 2023. Euro Surveill. 2023;28(49):2300606. 10.2807/1560-7917.ES.2023.28.49.2300606 38062942 PMC10831408

[7] Mira-Iglesias A Demont C López-Labrador FX Mengual-Chuliá B García-Rubio J Carballido-Fernández M Role of age and birth month in infants hospitalized with RSV-confirmed disease in the Valencia Region, Spain. Influenza Other Respir Viruses. 2022;16(2):328-39. 10.1111/irv.12937 34821055 PMC8818825

[8] European Centre for Disease Prevention and Control (ECDC). Core protocol for ECDC studies of COVID-19 vaccine effectiveness against hospitalisation with Severe Acute Respiratory Infection laboratory-confirmed with SARSCoV-2, version 1.0. Stockholm: ECDC; 2021. Available from: https://www.ecdc.europa.eu/sites/default/files/documents/Core-protocol-for-ECDC-studies-of-COVID-19-vaccine-effectiveness-against-hospitalisation-with-SARI.pdf

[9] Jackson ML Nelson JC . The test-negative design for estimating influenza vaccine effectiveness. Vaccine. 2013;31(17):2165-8. 10.1016/j.vaccine.2013.02.053 23499601

[10] Sullivan SG Feng S Cowling BJ . Potential of the test-negative design for measuring influenza vaccine effectiveness: a systematic review. Expert Rev Vaccines. 2014;13(12):1571-91. 10.1586/14760584.2014.966695 25348015 PMC4277796

[11] Ladhani SN Andrews N Parikh SR Campbell H White J Edelstein M Vaccination of Infants with Meningococcal Group B Vaccine (4CMenB) in England. N Engl J Med. 2020;382(4):309-17. 10.1056/NEJMoa1901229 31971676

[12] Parikh SR Andrews NJ Beebeejaun K Campbell H Ribeiro S Ward C Effectiveness and impact of a reduced infant schedule of 4CMenB vaccine against group B meningococcal disease in England: a national observational cohort study. Lancet. 2016;388(10061):2775-82. 10.1016/S0140-6736(16)31921-3 28100432

[13] Wildenbeest JG Billard MN Zuurbier RP Korsten K Langedijk AC van de Ven PM The burden of respiratory syncytial virus in healthy term-born infants in Europe: a prospective birth cohort study. Lancet Respir Med. 2023;11(4):341-53. 10.1016/S2213-2600(22)00414-3 36372082 PMC9764871

[14] Muñoz-Quiles C López-Lacort M Úbeda-Sansano I Alemán-Sánchez S Pérez-Vilar S Puig-Barberà J Population-based Analysis of Bronchiolitis Epidemiology in Valencia, Spain. Pediatr Infect Dis J. 2016;35(3):275-80. 10.1097/INF.0000000000000993 26658376

