# Supplementary material for: Early estimates of nirsevimab immunoprophylaxis effectiveness against hospital admission for respiratory syncytial virus lower respiratory tract infections in infants, Spain, October 2023 to January 2024
Source: Euro Surveill. 2024 Feb 8;29(6):2400046. doi: 10.2807/1560-7917.ES.2024.29.6.2400046 (PMC10853977; doi:10.2807/1560-7917.ES.2024.29.6.2400046)
Supplement: Supplement [file 24-00046_LACORT_SUPPLEMENT.pdf]

This supplementary material is hosted by Eurosurveillance as supporting information alongside the article [**Early estimates of nirsevimab immunoprophylaxis effectiveness against hospital admission for respiratory syncytial virus lower respiratory tract infections in infants, Spain, October 2023 to January 2024**] on behalf of the authors who remain responsible for the accuracy and appropriateness of the content. The same standards for ethics, copyright, attributions, and permissions as for the article apply. Eurosurveillance is not responsible for the maintenance of any links or email addresses provided therein.

Figure S1: LRTI distribution by age, region, nirsevimab immunization status and laboratory result for RSV

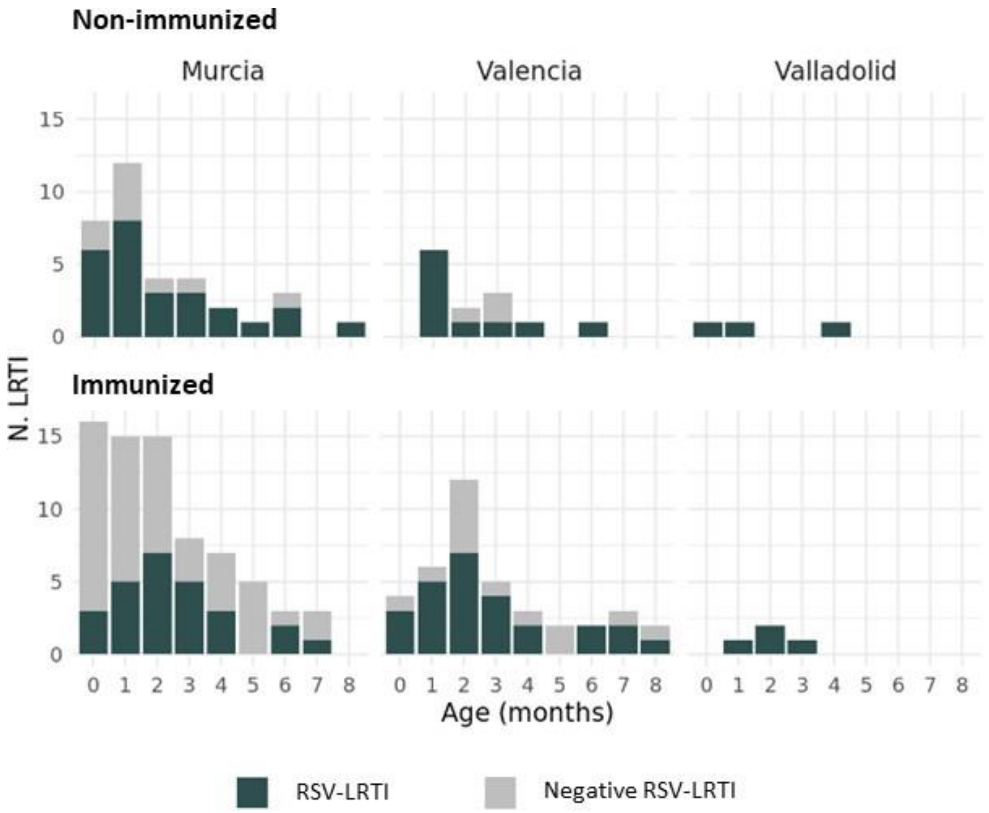

Distribution of Lower Respiratory Tract Infections (LRTI) positives and negatives for Respiratory Syncytial Virus (RSV) across different regions by age (in months), for non-immunized and immunized with nirsevimab.
